# High-throughput prediction of RNA, DNA and protein binding regions mediated by intrinsic disorder

**DOI:** 10.1093/nar/gkv585

**Published:** 2015-10-10

**Authors:** Zhenling Peng, Lukasz Kurgan

**Affiliations:** 1 Center for Applied Mathematics, Tianjin University, Tianjin, 300072, P.R. China; 2 Department of Electrical and Computer Engineering, University of Alberta, Edmonton, Alberta, T6G 2V4, Canada

## Abstract

Intrinsically disordered proteins and regions (IDPs and IDRs) lack stable 3D structure under physiological conditions *in-vitro*, are common in eukaryotes, and facilitate interactions with RNA, DNA and proteins. Current methods for prediction of IDPs and IDRs do not provide insights into their functions, except for a handful of methods that address predictions of protein-binding regions. We report first-of-its-kind computational method DisoRDPbind for high-throughput prediction of RNA, DNA and protein binding residues located in IDRs from protein sequences. DisoRDPbind is implemented using a runtime-efficient multi-layered design that utilizes information extracted from physiochemical properties of amino acids, sequence complexity, putative secondary structure and disorder and sequence alignment. Empirical tests demonstrate that it provides accurate predictions that are competitive with other predictors of disorder-mediated protein binding regions and complementary to the methods that predict RNA- and DNA-binding residues annotated based on crystal structures. Application in *Homo sapiens, Mus musculus, Caenorhabditis elegans* and *Drosophila melanogaster* proteomes reveals that RNA- and DNA-binding proteins predicted by DisoRDPbind complement and overlap with the corresponding known binding proteins collected from several sources. Also, the number of the putative protein-binding regions predicted with DisoRDPbind correlates with the promiscuity of proteins in the corresponding protein–protein interaction networks. Webserver: http://biomine.ece.ualberta.ca/DisoRDPbind/

## INTRODUCTION

Intrinsically disordered proteins and regions (IDPs and IDRs) lack stable 3D structure under physiological conditions in-vitro, actively participate in a wide repertoire of cellular functions and are relatively common in nature (1–3). Dozens of computational methods were developed to predict intrinsic disorder from the protein sequences (4–6).They were used to estimate the natural abundance of IDPs/IDRs and to investigate their functions (1,7–10). In particular, IDPs and IDRs were shown to be important for the protein–protein interactions (PPIs) and are enriched in the RNA- and DNA-binding proteins (1,11–16). Prediction of protein functions related to RNA binding (17,18), DNA binding (19,20) and PPIs (21,22) has generated strong interest in recent years. However, these predictions focus on the interactions that are extracted from crystal structures and thus which are primarily facilitated by ordered (structured) regions.

Similar studies for IDPs and IDRs also gain momentum. Recently, prediction of over 100 Gene Ontology (GO) annotations associated with disordered proteins was investigated (23). However, these predictions were performed at the whole protein level, were based on predicted disordered regions and assumed that the predicted IDRs contribute toward the GO annotations of the corresponding protein. The ANCHOR (24) and PepBindPred (25) methods that predict protein–protein binding residues located in IDRs and MoRFpred (26) and DISOPRED3 (27) methods that find short protein-binding regions (up to 25 consecutive residues) in IDRs that are involved in molecular recognition were also developed. These attempts suggest that functions of IDRs are predictable from the protein sequence. The DisProt database (28) includes over 800 functionally annotated IDRs, with about 500 that correspond to the disordered RNA-, DNA- and protein-binding; for convenience, we use the disordered RNA-, DNA- and protein-binding terms to denote the RNA-, DNA- and protein-binding located in IDRs. The availability of the annotated data, interest in these types of interactions and predictability of disorder-mediated functions motivate the development of our method DisoRDPbind. Our approach has the following four characteristics:

1. First attempt to predict multiple functions mediated by IDPs and IDRs. DisoRDPbind is the first method that predicts disordered RNA- and DNA-binding residues, and it also predicts disordered protein-binding residues.
2. High-throughput predictions. DisoRDPbind predicts an average size protein with 450 residues in two seconds on a modern desktop computer; this means that our method can be applied on the genomic scale.
3. Good predictive quality. DisoRDPbind is empirically shown to obtain good predictive performance using two independent (from a training data set) test data sets. Our method also provides accurate predictions when applied to find putative disordered RNA-, DNA- and protein-binding regions on four complete proteomes/genomes.
4. Complementarity to other predictors of DNA- and RNA-binding regions. DisoRDPbind's predictions are empirically shown to complement predictions of representative methods that were built using ordered DNA- and RNA-binding residues, i.e. using annotations based on crystal structures.

## MATERIALS AND METHODS

### Annotation of disordered RNA-, DNA- and protein-binding

The DisProt database (28) includes IDRs that were annotated with over 30 functional subclasses (29). We grouped multiple subclasses to define RNA-, DNA- and protein-binding. We define the disordered RNA-binding, DNA-binding and protein-binding by combining five, three and five functional subclasses, respectively (see Supplementary Table S1). Motivated by related works (30,31) we consider the disordered regions with at least 4 consecutive disordered residues.

### Benchmark data sets

We extracted all annotated proteins from release v5.6 of DisProt and removed those that contain IDRs that are annotated as ‘Unknown’ and ‘Disordered region is not essential for protein function’. Next, we clustered these proteins using CD-HIT (32) at 30% sequence similarity. We placed the entire protein clusters at random into one of the two data sets, the TRAINING data set with 315 proteins that we used to design the predictive model and the TEST114 data set that we utilized to benchmark the model. Consequently, proteins in the TEST114 set share below 30% sequence identity with the chains in the TRAINING data set. The TRAINING data set includes 2033, 5146 and 24290 disordered residues (from 14, 49 and 188 proteins, respectively) that are annotated with the RNA-, DNA- and protein-binding, respectively. TEST114 has 1271, 1420 and 6940 disordered residues (from 7, 13 and 60 proteins, respectively) with the RNA-, DNA- and protein-binding annotations, respectively.

We also considered proteins that were recently deposited in DisProt, between releases v5.6 and v6.01, to build the second test data set. We collected 36 proteins that constitute the TEST36 data set with 322, 948 and 2752 residues with the annotations of RNA-, DNA- and protein-binding, respectively. Supplementary Table S1 summarizes these three data sets. A given residue can be annotated with multiple functions and thus the total number of disordered residues annotated with a given function may be different than the sum of its functional subclasses. The three data sets are provided at http://biomine.ece.ualberta.ca/DisoRDPbind/.

We designed DisoRDPbind using cross-validation on the TRAINING data set, which was divided into the cross validation folds per sequence, i.e. entire sequences were placed into different folds. Moreover, we ensured that proteins in different cross validation folds share low (<30%) similarity by clustering with CD-HIT as described above.

### Assessment on the benchmark data sets

DisoRDPbind outputs real values that quantify propensity of each residue in the input protein sequence to participate in the DNA-, RNA- and protein-binding mediated by the intrinsic disorder. We assessed the predictive quality of these propensities using the receiver operating characteristic (ROC) curves. For each value of propensity *p* (between 0 and 1), the residues with propensity ≥ *p* are set as positives (binding), and all other residues are set as negatives (non-binding). For example, when predicting the disordered RNA-biding residues, the annotated RNA-biding residues (in a given data sets) are assumed as positives and all other annotated residues including the remaining disordered residues and all ordered residues are assumed as negatives. We computed the TP-rate = TP/(TP + FN) and the FP-rate = FP/(FP + TN) and used the area under the ROC curve (AUC) to quantify the predictive quality. TP (TN) is the number of true positives (negative), i.e. the number of correctly predicted positives (negatives), and FP (FN) denotes false positives (negatives), i.e. the number of negatives (positives) that were incorrectly predicted as positives (negatives). The TRAINING, TEST114 and TEST36 data sets are unbalanced and on average (over the three types of binding) about 9.2%, 6.9% and 10.1% of residues are annotated as binding, respectively. Thus, we reported the TP-rate at the FP-rate of 0.1 to assess the binary predictions (binding versus non-binding residue) of DisoRDPbind. This value quantifies fraction of correctly predicted binding residues when the fraction of incorrectly predicted non-binding residues is 0.1, which is similar to the number of positives. TP-rate at the FP-rate of 0.1 ranges from 0 to 1, where higher value (must be greater than 0.1 to improve over random prediction) implies better binary prediction.

We evaluated statistical significance of the differences in the AUC values between each considered predictor and DisoRDPbind. This assessment aims to investigate whether the results on a given data set are not biased by a subset of proteins by measuring if the predictive quality is consistent over different subsets of the data set. To accomplish that, we sampled the test data sets to accommodate for differences in predictive quality based on use of different benchmark data sets. Specifically, we randomly selected half of proteins from the TEST114 or TEST36 data set 10 times. Next, we compared AUC values of DisoRDPbind to a given considered method over the resulting 10 random subsets of each test data set. If the corresponding vectors of AUC values are normal, as tested using the Anderson–Darling test (33) test at the 0.05 significance, then we utilized *t*-test; otherwise we used the non-parametric Wilcoxon rank sum test (34). The differences with *P*-value < 0.05 are assumed statistically significant.

We also quantified the FP-rates on the non-binding regions for each binding type using the larger test data set, TEST114. We considered two types of the non-binding regions: disordered regions that have another functional annotation besides the ‘unknown’ and the currently tested RNA-, DNA-, or protein-binding annotations; and regions that are not annotated as disordered (i.e. ordered regions and the regions with no annotations in DisProt). For each type, we selected the non-binding regions at random to match their number and length with the number and sizes of the positive (disordered RNA-, DNA, or protein-binding) regions.

### Architecture of DisoRDPbind

**Figure 1.**
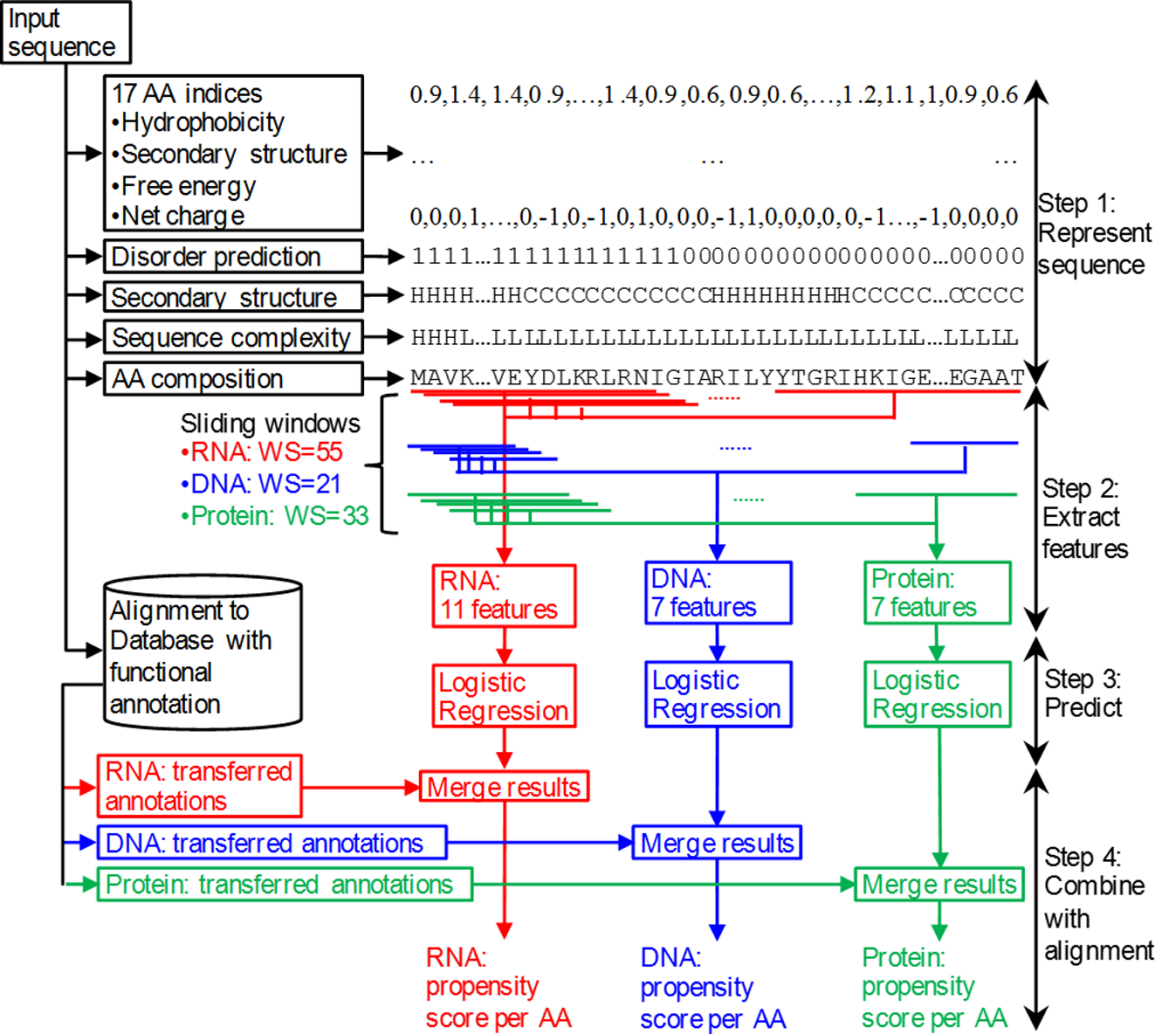
Architecture of DisoRDPbind.

Figure 1 shows the architecture of DisoRDPbind. Our method is based on a runtime efficient multi-layered design which is in line with a recent observation that specialized predictors with multiple inputs designed for specific functions are required (23). DisoRDPbind computes predictions in four steps. In step 1, a given input protein sequence is represented using a variety of physiochemical properties of amino acids (AAs), predicted intrinsic disorder and secondary structure, estimated value of sequence complexity, and the AA composition. In step 2, a set of 11, 7 and 7 numerical features (values) are generated from this information for the prediction of RNA-, DNA- and protein-binding residues, respectively. We considered a large number of features and performed empirical feature selection to obtain these small feature sets. In step 3, the selected features are inputted into three logistic regression models to predict the propensity score for each residues in the input sequence to participate in the disordered RNA-, DNA- and protein-binding. Inclusion of alignment was shown to be helpful in prediction of the PPIs including disordered proteins (35). Therefore, in step 4 we transfer the annotations of RNA-, DNA- and protein-binding based on sequence alignment generated by BLAST (36) using annotated chains in the corresponding training data set. These annotations are merged with the propensity scores generated by the regression to generate the final predictions.

### Feature extraction and selection

Several physicochemical properties of AAs, such as hydrophobicity, solvent accessibility, charge and free energy were successfully used to predict proteins with long disordered regions (37), disordered protein-binding residues (26) and RNA- and DNA-binding residues annotated using crystal structures (38,39). We utilized a wide range of AA indices that quantify various physicochemical properties of AAs. However, these AA indices may be redundant to each other or irrelevant to our prediction. Thus, we empirically selected a subset of non-redundant and relevant indices using the TRAINING data set. The original list of 531 amino acid (AA) indices from the version 9.1 of the AAindex database (40) was reduced to 159 indices that are predictive and dissimilar with each other (see Supplementary Materials for details). We considered the AA composition, sequence complexity and secondary structure based on the observations that IDRs are enriched in certain AAs (41), have low sequence complexity (42), and are biased in their secondary structure (43,44). Inclusion of putative disorder was shown to improve accuracy of prediction of functions related to signaling and molecular recognition (45) and was successfully utilized to predict disordered protein-peptide binding (26). To assure that DisoRDPbind is runtime-efficient we utilized disorder prediction generated by the fast IUPred method (46). We used three versions of IUPred that predict long and short disordered regions and globular domains.

The prediction for each residue in a given input chain uses information about the residue itself and its neighbors. We extracted information from a sliding window of size *ws* that is centered on the predicted residue to calculate features that are used as the inputs into the regression model. The use of the sliding window to calculate the features was inspired by previous related method (26,47). For the residues at the C or N-terminus of the sequence we reduce the window size on one side so it does not extend outside of the chain. We empirically derive the value of window size *ws* for each predicted function based on the size of the corresponding binding regions in the TRAINING data set. We set *ws* to the value of 20^th^ centile of the length of a given type of IDR, which translates into 55, 21 and 33 for the prediction of the disordered RNA-, DNA- and protein-binding residues, respectively. Motivated by recent work (26), we aggregate values of the numerical vectors to generate features by calculating the difference between an average value of the near neighbors, i.e. (*ws*-1)/2 residues in the middle of the sliding window, and remote neighbors, i.e. (*ws*-1)/4 residues at each termini of the sliding window. We utilize this aggregation to contrast the values calculated using positions in the chain that are close to the predicted residue against the values associated with residues in a wider neighborhood in a sequence. Detailed description of the features is given in the Supplementary Materials.

Some of the considered 398 features are redundant and/or irrelevant to the prediction of disordered RNA-, DNA- protein-binding residues. Thus, we performed empirical feature selection for each of the predicted functions in two steps that remove the irrelevant features (features that have poor predictive quality) and redundant features (correlated with other features). Details can be found in the Supplementary Materials. As a result, we selected 11, 7 and 7 features for the prediction of the disordered RNA-, DNA- and protein-binding residues, respectively. Only 17 AA indices are used to calculate the resulting selected features.

### Prediction model

Logistic regression is a probabilistic classification algorithm that was extensively used in related efforts including prediction of intrinsic disorder (6) and the ordered protein-RNA/-DNA/-protein interactions that were annotated using crystal structures (48–51). The popularity, short runtime and ability to provide the real-valued propensity motivated selection of this model. The regression coefficients for the selected features were estimated by using the ridge estimator based on the TRAINING data set for each of the three types of binding. The three real-valued scores that correspond to the predicted propensity of a given AA to participate in the disordered DNA-, RNA- and protein-binding that are outputted by the regression are merged with the outputs generated using sequence alignment with BLAST.

We used sequence alignment to transfer annotations of DNA-, RNA- and protein-binding from the TRAINING data set (or a training fold in case of the cross validation on the TRAINING data set). For a given query chain, the annotations are transferred (copied) for the similar positions in the alignment with the most similar sequence that has sufficiently high similarity quantified with the *e*-value. We chose 0.1 as the *e*-value cut-off, i.e. if the *e*-value < 0.1 then the aligned sequence(s) is regarded as sufficiently similar and the annotations are copied. This cut-off was chosen based on 4-fold cross validation on the TRAINING data set to maximize the average (over the predictions of the disordered RNA-, DNA- and protein-binding residues) ratio between TP-rate and FP-rate; use of ratio is motivated by the imbalanced nature of our data sets. Supplementary Figure S1 reveals that ratio for the *e*-value = 0.1 is the best. This ratio is higher than for the other *e*-values and the chosen *e*-value is lowest among comparable results (we secure similar ratio for the *e*-value = 1). During the transfer of annotations we set all the residues that are aligned to the binding residues to value of 1 and the remaining residues to 0. Consequently, the annotations transferred using BLAST are binary.

Empirical results where we transferred the annotations using alignment with BLAST on the 4-fold cross validation on the TRAINING data set with the *e*-value cut-off of 0.1 show that BLAST nearly perfectly predicts negatives (i.e. TN/N > 99%) and captures a small number of true positives (i.e. TP-rate < 5%). This conservative prediction (small number of high quality predictions of binding residues) is merged with the prediction from the regression as follows. If a given residue is annotated with a given disordered function by the alignment then its propensity score is set to (1+*p_i_*)/2, where *p_i_* is the propensity score produced by the regression model and *i* denotes a particular function: disordered DNA-, RNA- or protein-binding; otherwise we use the prediction generated by the regression model. This increases values of the propensities generated by the regression for residues that were also predicted as binding by the alignment. The AUCs of the model that combines regression with BLAST on the 4-fold cross validation on the TRAINING data set (i.e. only the sequences from the training folds in the cross-validation are used to build the regression model and as the data set for the alignment) are 0.75, 0.7 and 0.63 for the prediction of the disordered RNA-, protein- and DNA-binding residues. The final model that is available as the webserver and which we assessed on the two test data set combines the results of the regression model built on the TRAINING data set and alignment with BLAST against the proteins from the TRAINING data set.

### Whole proteome data sets

We used complete proteomes of four popular eukaryotic model organisms collected from release 2013_04 of the UniProt database (52) to apply and evaluate DisoRDPbind on the genomic scale. We removed protein fragments based on the term ‘Fragment’ in the subsection ‘Sequence status’. The resulting proteomes include 42426, 33181, 25159 and 19656 proteins for *H. sapiens, M. musculus, C. elegans* and *D. melanogaster*, respectively. We compared our predictions against the known DNA and RNA-binding proteins in these proteomes that were annotated based on several large databases including gene ontology (GO) terms (53) in UniProt, RBPDB (54) for the RNA-binding proteins, and animalTFDB (55) for the DNA-binding proteins. Considering the hierarchical structure of GO, we defined the RNA (DNA) binding by collecting the GO term RNA (DNA) binding itself and all of its children connected by ‘is_a’ relation. We collected 3298 RNA-binding proteins (GO_RNA) and 7880 DNA-binding proteins (GO_DNA) across these four proteomes. By mapping accession number of proteins from UniProt into RBPDB and animalTFDB resources, we obtained annotations of 1014 RNA-binding and 4089 DNA-binding proteins, respectively, over the four organisms. Moreover, we also extracted 1870 and 803 curated RNA- and DNA-binding proteins, respectively, in *H. sapiens* and *M. musculus* from recent literature (56–58). We denoted these two data sets as DB_RNA and DB_DNA, respectively.

We utilized the latest integrated database of PPI networks, mentha (59), for the assessment of the prediction of the disordered protein-binding regions on the genomic scale. We mapped proteins from UniProt into mentha and obtained an average of 21.4, 6.7, 5.2 and 7.3 interactions per protein for the 14547, 8006, 5005 and 8096 proteins from *H. sapiens, M. musculus, C. elegans* and *D. melanogaster*, respectively. Finally, we collected eukaryotic linear motifs (ELMs), short regions and perform regulatory functions via PPIs (60), to assess whether they are predicted by our method. ELMs from *H. sapiens* and *M. musculus* were extracted from the ELM database (60); we removed entries tagged as ‘false positive’ and those that were not verified experimentally. Consequently, we collected 1448 ELMs from 952 proteins over *H. sapiens* and *M. musculus* proteomes.

For convenience, we use the source database name to represent the corresponding subset of proteins selected for the four organisms. Supplementary Table S2 summarizes the eight data sets: GO_RNA, GO_DNA, RBPDB, animalTFDB, DB_RNA, DB_DNA, mentha and ELM. We also predicted intrinsic disorder on these data sets using consensus of five methods and computed the disorder content (fraction of disordered residues) for each species and protein; see details in the Supplementary Materials and summary of results in the Supplementary Table S2.

### Assessment at the whole proteome level

Since proteins in the whole proteome data sets are annotated with a given function per sequence, we define the disordered RNA-, DNA- and protein-binding proteins predicted by DisoRDPbind from our residue-level predictions as follows. First, we binarized the predicted propensities using the default cut-off of 0.5. We assume a given protein as the disordered RNA-, DNA- and/or protein-binding protein if it has at least one predicted disordered RNA-, DNA- and/or protein-binding regions composed of at least 4 consecutive residues, respectively. This is consistent with prior works that assume that IDRs include at least 4 consecutive disordered residues (30,31).

To evaluate prediction of the disordered RNA-binding (DNA-binding) proteins for a given organism we calculated overlap between the set of the predicted disordered RNA-binding (DNA-binding) proteins and the proteins from RNA-binding data sets: GO_RNA, RBPDB and DB_RNA (from the DNA-binding data sets: GO_DNA, animalTFDB and DB_DNA). We assessed statistical significance of this overlap by comparing it to an overlap with a randomly generated set of proteins. First, we selected at random half of the predicted RNA-binding (DNA-binding) proteins 10 times and estimated their overlap with the GO_RNA, RBPDB and DB_RNA (GO_DNA, animalTFDB, or DB_DNA). Next, we selected at random the same number of proteins, when compared to the number of predicted RNA-binding (DNA-binding) proteins, from a given complete proteome 10 times and computed their overlap with the same RNA-binding (DNA-binding) data sets. We compared the ten corresponding values of overlap to find whether the overlap of our predictions is significantly higher than a baseline defined based on overlap with the random set of proteins. If both vectors of the overlap values are normal, as tested using Anderson–Darling test at the 0.05 significance, then we utilized *t*-test; otherwise we used the non-parametric Wilcoxon rank sum test. The differences with *P*-value < 0.05 are assumed statistically significant.

Moreover, the novel RNA-binding (DNA-binding) proteins predicted by DisoRDPbind that do not overlap with the known RNA-binding (DNA-binding) proteins from GO_RNA, RBPDB and DB_RNA (GO_DNA, animalTFDB and DB_DNA) were further analyzed. We investigated their cellular localization based on the GO annotations. Our aim was to find out whether their localizations are similar to the localizations that are significantly associated with the known RNA-binding (DNA-binding) proteins. We found that 72%, 57%, 44% and 32% proteins from *M. musculus, H. sapiens, D. melanogaster* and *C. elegans* are annotated with GO annotations of cellular component (i.e. localization), respectively (Supplementary Table S2). Thus, we performed this analysis in *M. musculus* due to the low coverage of these annotations in the other organisms. We first determined the cellular localizations that are significantly associated with the known RNA-binding (DNA-binding) proteins. Specifically, we selected at random half of the known RNA-binding (DNA-binding) proteins 10 times and quantified their cellular localizations by computing a fraction of proteins with each of these annotations. Next, we selected at random the same number of proteins from the entire *M. musculus* proteome 10 times and quantified their cellular localizations in the same way. We assessed significance of the differences between the corresponding two vectors of fractions (for the ‘known’ and ‘random’ proteins) by following the above mentioned procedure to assess the overlap. This resulted in a list of cellular localizations that are significantly associated with the known RNA-binding (DNA-binding) proteins. Next, we investigated the significance of an overlap between these localizations and the localizations of novel RNA-binding (DNA-binding) proteins predicted by DisoRDPbind, following the above mentioned procedure to assess the overlap. We removed the localizations with low counts, i.e. less than 2% of the total count of all localization annotations in a given protein set, to avoid spurious measurements. We assert that our predictions are accurate if this overlap is statistically significant with *P*-value < 0.05.

Since most proteins interact with other protein(s), we cannot directly validate the prediction of protein-binding using the abovementioned procedure. However, we use the observation that hub proteins are enriched in IDRs (12,61). Therefore, we investigated relation between the promiscuity of a given protein (number of its proteins partners in the corresponding PPI network) and the number of its predicted disordered protein-binding regions to assess the predictive quality of DisoRDPbind at the whole proteome level. This relation was quantified with the Pearson Correlation Coefficient (PCC) between the average number of partners for proteins with a given number of predicted disordered protein-binding regions and this number of regions. We assert that our predictions of disordered protein-binding regions are likely correct if the PCC value is relatively high and positive. We analyzed statistical significance of this PCC value by comparing it to a PCC value obtained using the average number of partners for a set of proteins with randomized number of the predicted regions. First, we selected at random half of the proteins for each number of predicted disordered protein-binding regions in the mentha data set 10 times and computed the PCC between the number of their predicted regions and their average promiscuity defined in mentha. We repeated the computation of PCC 10 times using randomly selected sets of proteins of the same size as the number of proteins with a given number of predicted regions and correlating this ‘randomized’ number of regions with their average actual promiscuity extracted from mentha. Finally, we computed statistical significance of the difference between these two vectors of 10 PCC values using the procedure described to assess the overlap for the prediction of the disordered RNA- and DNA-binding proteins.

## RESULTS AND DISCUSSIONS

### Comparative evaluation of DisoRDPbind

Since there are no other methods that predict disordered RNA- and DNA-binding residues, we empirically compared predictive quality of DisoRDPbind with representative (latest and accurate) sequence-based methods that predict ordered RNA- and DNA-binding; selection of these methods is explained in the Supplementary Materials and in Supplementary Table S3. They include BindN+ (62) and RNABindR 2.0 (38) for the RNA-binding, and BindN+ and DNABR (63) for the DNA-binding. We also compared with the three predictors of the disordered protein–protein interacting residues: MoRFpred (26), DISOPRED3 (27) and ANCHOR (24); we did not include PepBindPred (25) due to the relatively long runtime required for the molecular dynamics simulations used by this method.

**Figure 2.**
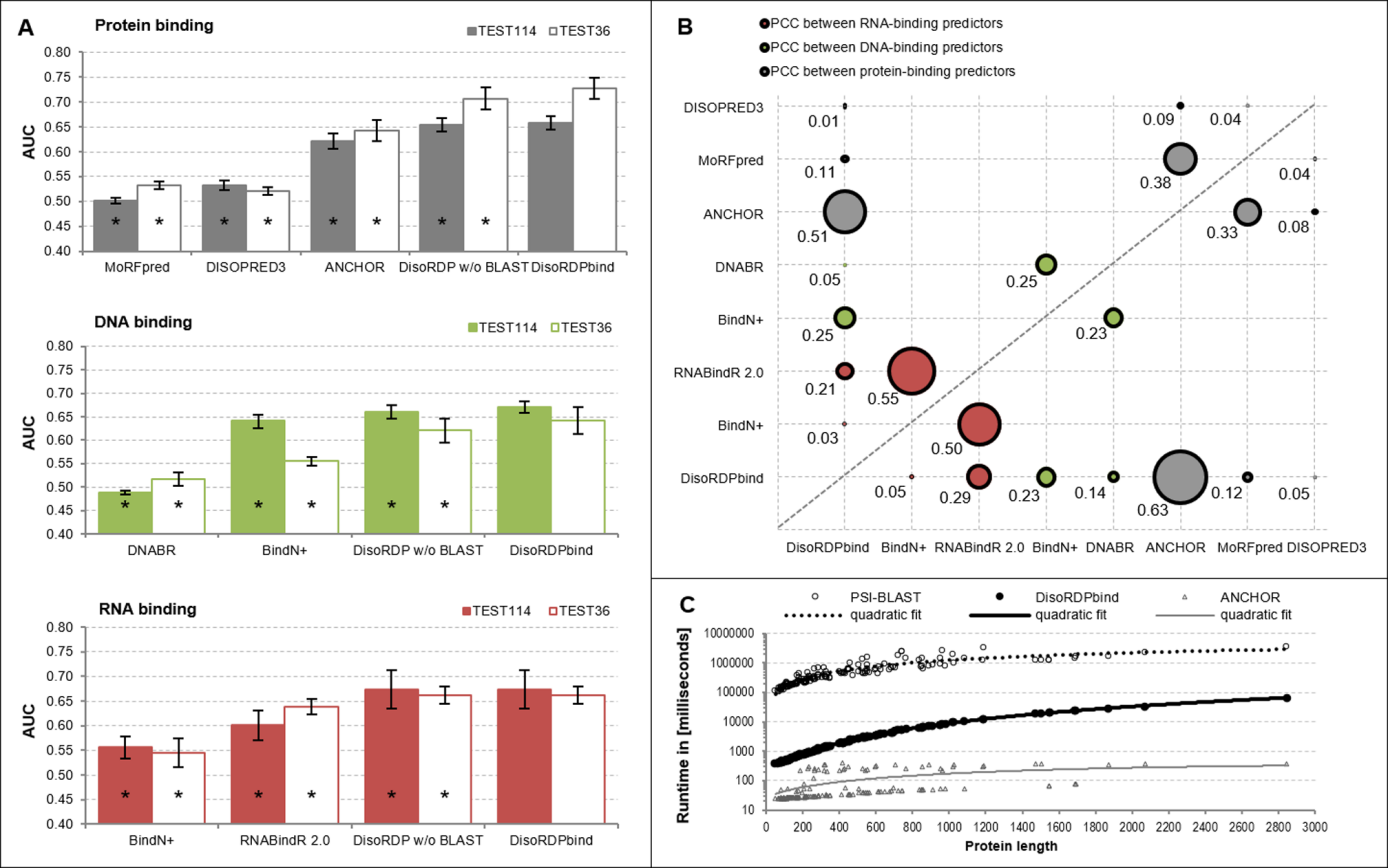
Empirical assessment of the prediction of the disordered RNA-, DNA- and protein-binding residues. (**A**) predictive performance measured with AUC calculated per residue and significance of differences in AUC values when comparing DisoRDPbind with other methods on two benchmark data sets: TEST114 (solid bars) and TEST36 (hollow bars); * means that AUC of a given method is statistically significantly lower than AUC of DisoRDPbind at *P*-value < 0.05; statistical significance was assessed over 10 random subsets with half of proteins from a given test set; error bars show the corresponding standard errors (details in Online Methods). ‘DisoRDP w/o BLAST’ denotes DisoRDPbind without the use of the BLAST-based alignment. (**B**) PCC values between the propensity scores generated by the pairs of RNA- (red dots), DNA- (green dots) and protein- (black dots) binding predictors listed on the *x* and *y*-axes; results for the TEST114 and TEST36 data sets are shown above and below the dashed diagonal line, respectively; dot sizes are proportional to the corresponding PCC value that are shown next to the dots. (**C**) Relation between length of protein chains (*x*-axis) and the runtime (*y*-axis in the logarithmic scale) computed for proteins from the TEST114 and TEST36 data sets using a modern desktop; we include DisoRDPbind (solid circles), ANCHOR (hollow triangles), and one iteration of PSI-BLAST (hollow circles); the solid black, solid gray and dotted black lines represent the quadratic fit for DisoRDPbind, ANCHOR and PSI-BLAST, respectively.

Figure 2A summarizes results on two benchmark data sets, TEST114 and TEST36. DisoRDPbind obtains the area under the ROC curve (AUC) values ranging between 0.62 and 0.72, depending on the benchmark data sets and the predicted function. These AUCs are higher across predictions of DNA-, RNA- and protein-binding residues on both benchmark sets when compared with the other methods; the improvements are statistically significant at *P*-value < 0.05. Moreover, the ROC curves of DisoRDPbind are above the ROC curves of the other methods on both data sets (Supplementary Figure S2). The lower predictive performance of the other DNA- and RNA-binding predictors can be explained by their focus on the structured interactions. MoRFpred predict only short binding regions (up to 25 residues) used in recognition, as opposed to DisoRDPbind that also predicts longer protein binding regions.

The average, over the two benchmark sets, TP-rate (fraction of correctly predicted binding residues) of DisoRDPbind computed at the FP-rate (fraction of incorrectly predicted non-binding residues) of 0.1 equals 0.27, 0.25 and 0.24 for the prediction of the DNA-, protein- and RNA-binding residues, respectively (Supplementary Figure S2). This means that the TP-rate is between 2.4 and 2.7 times higher than the corresponding FP-rate. DisoRDPbind secures TP-rate of 0.4 at the average, over the two benchmark data sets, FP-rate of 0.19, 0.21 and 0.23 for the protein, DNA and RNA-binding, respectively. Inclusion of the alignment into DisoRDPbind provides only slight improvements for the prediction of the disordered DNA- and protein-binding residues (two right-most sets of bars in Figure 2A). This demonstrates that most of the predictions are generated by the regression models. In fact, one average over the two benchmark sets BLAST finds only 4.3% and 1.1% of the DNA- and protein-binding residues, and no RNA-binding residues. This is expected given the low similarity between our benchmark data sets and the training proteins that are used to perform alignment. However, these small improvements are statistically significant, which means that addition of BLAST provides slight but consistent (over each test set) increase in the predictive performance. This is further supported by the fact that the ROC curves of DisoRDPbind are slightly and consistently above the ROC curves of DisoRDPbind without BLAST for the prediction of the disordered DNA- and protein-binding residues on TEST114 and TEST36 (Supplementary Figure S2).

We assessed predictive performance of DisoRDPbind on two types of non-binding regions extracted from TEST114: disordered regions annotated with functions excluding a binding type that we evaluate, and regions that are not located in the annotated disordered regions (Supplementary Figure S3). We quantified FP-rate values since these are non-binding regions. DisoRDPbind generates FP-rates of 0.07, 0.09 and 0.11 (0.06, 0.12 and 0.01) for the prediction of the DNA-, protein- and RNA-binding, respectively, on the regions that exclude the annotated disordered regions (disordered regions that exclude the predicted type of binding). These are acceptable levels of FP-rates given that the binary predictions of DisoRDPbind were designed to provide the PF-rate of 0.1 on the TRAINING data set (significance of this PF-rate is explained in Materials and Methods). The other methods obtain comparable levels of the FP-rates, ranging from 0.02 to 0.14 for the prediction of disordered RNA-binding, 0.01 to 0.14 for the DNA-binding and 0.05 to 0.12 for the protein-binding.

We also measured Pearson correlation coefficient (PCC) between predictions of DisoRDPbind and the other considered methods to investigate their similarity (Figure 2B). The low PCCs < 0.3 of DisoRDPbind with BindN+ and with RNABindR reveal that DisoRDPbind's predictions of the RNA-binding residues are different from (complementary to) the predictions from these two methods. The same is true for the prediction of the DNA-binding residues when comparing DisoRDPbind with BindN+ and with DNABR. Moreover, DisoRDPbind's predictions of the disordered protein-binding residues are characterized by relatively high PCC > 0.5 with the outputs of ANCHOR and low correlation with MoRFpred and DISOPRED3. This is also expected since MoRFpred and DISOPRED3 predict the short binding regions while both DisoRDPbind and ANCHOR predict generic disordered protein-binding regions. To sum up, DisoRDPbind's predictions are different from the outputs of the DNA- and RNA-binding predictions that are focused on the ordered regions and improve over the existing predictors of the disordered protein-binding regions.

### Evaluation of runtime

We compared runtime of DisoRDPbind with ANCHOR and with one iteration (*j* = 1) of PSI-BLAST (36) against the nr database (Figure 2C). The latter estimates a lower bound of the runtime of the other predictors, such as BindN+, RNABindR, DNABR, MoRFpred and DISOPRED3, which use PSI-BLAST. Although DisoRDPbind is slower than ANCHOR by up to two folds, it provides prediction of the three considered functions at the same time. DisoRDPbind is over 150 times faster than the one round of PSI-BLAST. Depending on the chain length, prediction for one protein takes between 0.3 s and 1 min using a modern desktop computer. The runtimes are characterized by a quadratic increase with the chain size. PCCs between the quadratic fit and the measured runtime for DisoRDPbind and PSI-BLAST equal 1 and 0.83, respectively. The total runtime of DisoRDPbind over the complete *H. sapiens* proteome (42 426 chains) was 45 h, compared to the quadratic fit-based estimates of 43 h and 261 days for DisoRDPbind and one round of PSI-BLAST, respectively.

### Validation on the whole proteomes

**Figure 3.**
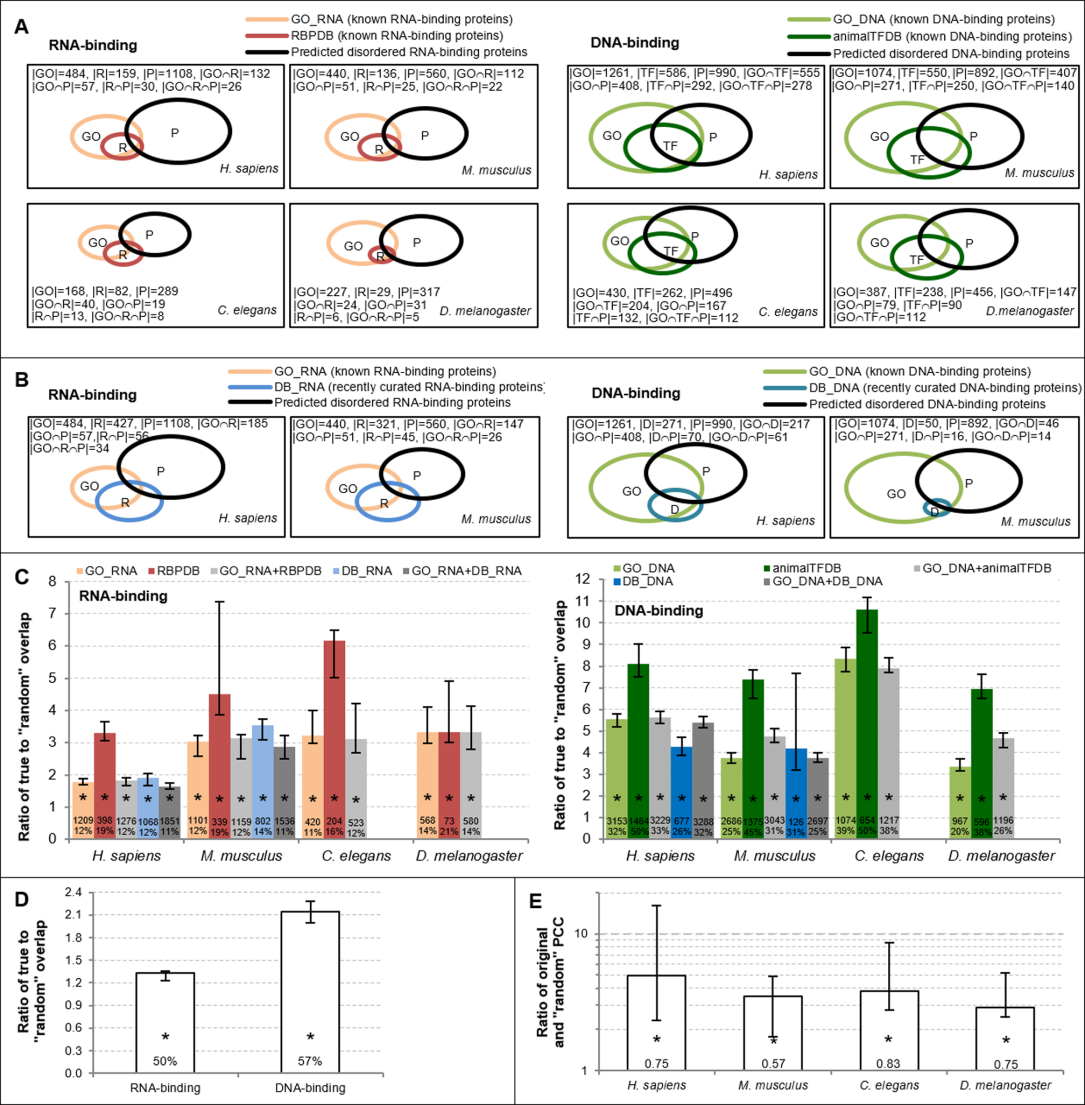
Evaluation of predictions of the disordered RNA-, DNA- and protein-binding in the *H. sapiens, M. musculus, C. elegans* and *D. melanogaster* genomes. (**A**) Venn diagrams of the overlap between the disordered RNA-binding (DNA-binding) proteins predicted by DisoRDPbind and the known binding proteins collected from the GO_RNA (GO_DNA) and RBPDB (animalTFDB) data sets, respectively. (**B**) Venn diagrams of the overlap between the disordered RNA-binding (DNA-binding) proteins predicted by DisoRDPbind and the known binding proteins collected from the GO_RNA (GO_DNA) and from recently curated RNA-binding (DNA-binding) protein data set DB_RNA (DB_DNA), respectively. The area of the rectangles corresponds to 40% of size of a given proteome; The counts of proteins in a given data set and intersections of the data sets are given inside the corresponding rectangles; (**C**) Median ratio between the actual overlap between the RNA-binding (DNA-binding) proteins predicted by DisoRDPbind and proteins annotated in the GO_RNA, RBPDB and DB_RNA (GO_DNA, animalTFDB and DB_DNA), and the overlap of the proteins from these databases with a randomly chosen set of proteins. The median ratio is over 10 repetitions with half of the data; error bars are 30% and 70% centiles; the number of chains in a given database and percentage of overlap with the predictions of DisoRDPbind are given inside the bars; * means that the difference between the two values of overlap is statistically significant at *P*-value < 0. 0005. (**D**) Median ratio (over 10 repetitions with half of the data; error bars are 30% and 70% centiles) between the actual overlap between the cellular localizations of novel putative RNA (DNA) binders and the localizations that are significantly associated with the proteins known to bind RNA from GO_RNA, RBPDB and DB_RNA (known to bind DNA from GO_DNA, animalTFDB and DB_DNA), and the overlap in cellular localizations of the proteins from these databases with a randomly chosen set of proteins. This analysis was done in *M. musculus* since annotation of localizations in other genomes were not sufficiently complete. The percentage of the overlap with the predictions of DisoRDPbind is given inside the bars;* denotes that the difference between the two values of overlap is statistically significant at *P*-value < 0.05. (**E**) Relation between the promiscuity of proteins in PPI networks collected from mentha and the number of the disordered protein-binding regions predicted with DisoRDPbind. The relation was quantified with Pearson correlation coefficient (PCC) that is show inside the bars. Bars shows median ratio (over 10 repetitions with half of data; error bars are 30% and 70% centiles) in logarithmic scale between these PCC values and the ‘random PCC’ where the promiscuity values were shuffled; * denotes that the difference between the two values of PCC is statistically significant at *P*-value < 0.05.

We applied DisoRDPbind to perform predictions for four complete proteomes: *H. sapiens, M. musculus, C. elegans* and *D. melanogaster*. DisoRDPbind predicted 2769 (2475), 1041 (2231), 722 (1241) and 792 (1140) proteins as the disordered RNA-binding (DNA-binding) in human, mouse, worm and fly, respectively. The predicted RNA-binding (DNA-binding) proteins have on average 32–37% (30–40%) of disordered residues. This is substantially higher than the average fraction of disordered residues in these four species (Supplementary Table S2), which is consistent with prior results (1). We further assessed these predictions by quantifying an overlap between the disordered RNA- and DNA-binding proteins predicted by DisoRDPbind and the native RNA- and DNA-binding proteins from Gene Ontology (53) (GO_RNA and GO_DNA sets), RBPDB(54), animalTFDB(55) (Figure 3A) and binding proteins collected from recent literature: DB_RNA and DB_DNA sets (Figure 3B) (we could not derive a precise and proportional Venn diagram when considering all four data sets); see details in Materials and Methods and Supplementary Table S2. Depending on the organism and the data set, between 11% and 21% of known RNA-binding proteins (i.e. 11–14% from GO_RNA, 16–21% from RBPDB and 12–14% from DB_RNA), and between 20% and 50% of known DNA-binding proteins (i.e. 20–39% from GO_DNA, 38–50% from animalTFDB and 26–31% from DB_DNA) were predicted by DisoRDPbind. Our analysis (Figure 3C) reveals that this overlap is between 1.6 and 6.2 higher (depending on the organism and database) for the RNA-binding and between 3.5 and 10.6 times higher for the DNA-binding when compared with the overlap for a random set of the same number of proteins as we predicted. These differences are statistically significant and they suggest that our predictions are plausible. Figure 3A and B show that majority of the predicted disordered RNA-binding (DNA-binding) proteins are novel putative binders, i.e. not included in GO_RNA, RBPDB and DB_RNA (GO_DNA, animalTFDB and DB_RNA). These novel binders have much lower levels of functional annotations (43% versus 79% for known binding proteins annotated in GO; see Supplementary Table S4), which motivates our predictions. We analyze an overlap between the annotations of their cellular localizations and the cellular localizations that are significantly associated with the known binding proteins (see Materials and Methods for details). We performed this analysis in *M. musculus*, which has by far the most complete annotations among the four species for the putative binders (see Supplementary Table S4). About 50% (57%) of the cellular localization annotations of the novel putative RNA (DNA) binders overlap with the localization of the known binders. This overlap is significantly higher than the overlap for a randomly chosen set of proteins, with the increase by 1.35 and 2.15 times for the RNA-binding and DNA-binding, respectively (Figure 3D). This suggests that the novel putative binders could be correctly predicted.

We also assessed the DisoRDPbind's prediction of the disordered protein-binding on the four proteomes. We analyzed relation between the promiscuity of a given protein, i.e. number of its protein partners in the PPI network collected from the latest integrated database mentha (59), and the number of its predicted disordered protein-binding regions (see Materials and Methods for details). The corresponding PCC values are 0.75, 0.57, 0.83 and 0.75 for the proteins from *H. sapiens, M. musculus, C. elegans* and *D. melanogaster*, respectively (Figure 3D). We assessed whether this observation is statistically significant by comparing these correlations with the PCCs obtained when using proteins with randomized number of predicted disordered protein-binding regions (see Materials and Methods for details). Figure 3D shows that the original correlations are at least 2.7 times higher than the ‘random’ correlations; this increase is statistically significant. This suggests that proteins with more predicted disordered protein-binding regions generally interact with more protein partners, which is consistent with prior results that hub proteins (that interact with at least 10 partners) are enriched in disorder compared to the proteins that interact with one partner (12,61).

We investigate whether the disordered protein-binding regions predicted by DisoRDPbind intersect with the ELMs, which are intimately involved in the PPIs (60) (see Materials and Methods for details). We assume that they overlap if at least one residue is located in both of these two regions. Our analysis shows that 568 and 118 ELMs from *H. sapiens* and *M. musculus*, respectively, are located in the disordered regions, and 95–97% of them overlap with the disordered protein-binding regions (see Supplementary Table S2). This further supports our claim that DisoRDPbind provides accurate predictions of the disordered protein-binding.

## CONCLUSIONS

DisoRDPbind offers good predictive performance and short runtime, which facilitates genome-scale applications. Its outputs complement predictions of representative methods that were built using structured DNA- and RNA-binding residues. Based on the analysis of genome-scale predictions, our method can be used to find new DNA- and RNA-binding proteins. Predictions of disordered protein-binding residues generated by DisoRDPbind are characterized by strong correlations, better predictive performance and higher runtime when compared with the closest ANCHOR method. We confirm that promiscuity of proteins in PPI networks is correlated with the number of their disordered protein-binding regions and demonstrate that 95% of ELMs that are located in the disordered regions overlap with our predictions.

## SUPPLEMENTARY DATA

Supplementary Data are available at NAR Online.

SUPPLEMENTARY DATA
