# Supplementary material for: High-throughput prediction of RNA, DNA and protein binding regions mediated by intrinsic disorder
Source: Nucleic Acids Res. 2015 Oct 10;43(18):e121. doi: 10.1093/nar/gkv585 (PMC4605291; doi:10.1093/nar/gkv585)
Supplement: SUPPLEMENTARY DATA [file supp_43_18_e121__index.html]

High-throughput prediction of RNA, DNA and protein binding regions mediated by intrinsic disorder — High-throughput prediction of RNA, DNA and protein binding regions mediated by intrinsic disorder — SUPPLEMENTARY DATA 

# High-throughput prediction of RNA, DNA and protein binding regions mediated by intrinsic disorder

## SUPPLEMENTARY DATA

- SUPPLEMENTARY DATA
