# Supplementary material for: High-throughput prediction of RNA, DNA and protein binding regions mediated by intrinsic disorder
Source: Nucleic Acids Res. 2015 Oct 10;43(18):e121. doi: 10.1093/nar/gkv585 (PMC4605291; doi:10.1093/nar/gkv585)
Supplement: SUPPLEMENTARY DATA [file supp_gkv585_nar-00184-met-z-2015-File005.pdf]

Supporting materials for
"HIGH-THROUGHPUT PREDICTION OF RNA, DNA, AND PROTEIN BINDING REGIONS MEDIATED BY INTRINSIC DISORDER"

Zhenling Peng[1,2] and Lukasz Kurgan[1,*]

[1] Department of Electrical and Computer Engineering, University of Alberta, Edmonton, Alberta, T6G 2V4, Canada

[2] Center for Applied Mathematics, Tianjin University, Tianjin, China

* To whom correspondence should be addressed. Tel: 1-780-492-5488; Fax: 1-780-429-1811; Email: lkurgan@ece.ualberta.ca

MATERIALS AND METHODS

Selection of Amino Acid Indices

We collected 531 amino acid (AA) indices from the version 9.1 of the AAindex database (1-4), after removing 13 AA indices with unknown values. We collected disordered segments with a given functional annotations (set A) and all other segments (including disordered and ordered segments; set B) from the TRAINING dataset. Next, we randomly selected 40% of segments from set A and the same number of segments from set B (the choice of 40% is motivated by the size of the annotation sets to assure that they can be matched), and considered a given AA index by averaging the corresponding numerical values in all segments in each of the two sets. This was repeated ten times for a given AA index. Consequently, we obtained two vectors of ten averages. We evaluated significance of the differences between these two vectors. If the measurements are normal, as tested with the Anderson-Darling test (5) at the 0.05 significance, then we utilized the *t*-test; otherwise we used the non-parametric Wilcoxon rank sum test (6). Since we considered the disordered DNA-, RNA- and protein-binding, we obtained three *p*-values for each AA index. We averaged these three *p*-values for each AA index and asserted that a lower average indicates stronger relations between the corresponding AA index and the disordered DNA-, RNA- and protein-binding regions. The averages were used to rank the AA indices in the ascending order. Next, to remove redundant indices, we selected the top ranked index to initialize the set of selected indices and added a subsequently ranked index if its PCC (7,8) with each AA index that is already in the selected set is < 0.75; otherwise we rejected a given index since it is similar (redundant) (i.e., has PCC ≥ 0.75) with the already chosen indices. The entire list of ranked indices was scanned once. In total, 159 AA indices were selected.

Feature Extraction and Selection

We utilize a sliding window to represent information used to perform prediction of the central (in the center of the window) residue; this was done for each residue in the input sequence. The window sizes *ws* were set to 55, 21, and 33 for the disordered RNA-, DNA- and protein-binding residues, respectively. Each position/residue in the input sequence is represented by the following six sets of features:

1. Amino acid (AA) composition, which is defined as the fraction of a given AA type within the sliding window (20 features).
2. Features based on sequence complexity generated by SEG algorithm (9) (7 features). Within a given sliding window, we calculated the fraction of AAs in low complexity regions (1 feature), and the average/maximum/minimum length of the low/high complexity segments that is normalized by dividing the number of corresponding

complexity segments (2*3 = 6 features). If there is no low (high) complexity region in the sliding window then we set the normalized average/maximum/minimum length of the low (high) complexity segments to 0.

3. Features based on the secondary structure predicted with the fast PSIPRED (without using PSI-BLAST) (10) (12 features). Using the sliding window, we computed the fraction of AAs in helix, strand and coil confirmations, respectively (3 features), and the average/maximum/minimum segments length for a given type of the secondary structure that is normalized by dividing by the number of segments of the corresponding type (3*3 = 9 features).
4. Features based on the putative disorder and globular domain that are predicted with IUPred (11,12) (11 features). Based on IUPred prediction for long and short disordered segments and globular domains, we computed the disorder content (i.e., fraction of disordered residues) and the fraction of AAs in globular domains (3 features), the normalized average/maximum/minimum length of disordered segments with at least 4 residues (3*2 = 6 features), and the average of the two raw propensity values generated by IUPred (2 features).
5. Features based on the selected AA indices (i.e., physicochemical properties of AAs) (159 features). We averaged the numerical values of a given AA index in the sliding window.
6. Aggregated features that consider difference between an average value of particular property of the near neighbors, i.e., (*ws*-1)/2 residues in the middle of the sliding window, and remote neighbors, i.e., (*ws*-1)/4 residues at each termini of the sliding window (189 features). We compute these differences for the values of AA composition (20 features), the fractions of residues in low complexity regions (1 features) and in a given type of secondary structure (3 features), the content of predicted disordered and structured residues (3 features) and the average of predicted propensity scores (3 features) using IUPred's outputs, and the average of the selected AA indices to represent the physicochemical properties (159 features).

In total, we considered 398 features.

A subset of predictive and non-redundant features was empirically selected from the considered feature set in two steps. Step 1 removes the irrelevant features, i.e., features that have poor predictive quality. We analyzed the strength of relations between values of a given feature and the annotation of disordered RNA-, DNA-, and protein-binding residues in the TRAINING dataset; the relation was quantified with the point-biserial correlation (PBC) (13). If the strength of the relation for a given feature is low, i.e., |PBC value| < 0.02, then we removed this feature. In step 2, we further filtered out the redundant and irrelevant features using wrapper feature selection (14) utilizing logistic regression as the classifier (prediction model). This step maximizes predictive quality measured with AUC by varying feature sets; the predictions were done using 3+1-fold cross validation on the TRAINING dataset. This type of cross validation was introduced in Ref. (15) to reduce overfitting. In the 3+1-fold cross validation, we fixed one of the four cross validation folds as a test dataset and the remaining three folds are used to perform three-fold cross validation. We tested each predictive model twice: based on the three-fold cross validation and based on the fourth test fold. The selection process starts by ranking all features in the descending order of their absolute PBCs computed on the TRAINING dataset. The set of selected features is initialized with the top ranked feature, which has consistent sign and at least 0.02 absolute PBC values across all four folds. We added a subsequently ranked feature to the set of selected features if it satisfies the same condition and if this addition improves AUC on both the three-fold cross validation and the independent fourth test fold by at least 0.001. We scanned the ranked feature list once.

Consensus-based Disorder Prediction on Whole Proteomes

We applied two predictors: IUPred (11,12) and ESpritz (16), to obtain putative disordered residues that were used to analyze our predictions. These methods were shown to provide good predictive quality (12,17,18) and are computationally efficient. Two versions of IUPred were designed for the predictions of long and short disordered segments, respectively. ESpritz has three versions that consider disorder annotations based on the X-ray crystal structures, nuclear magnetic resonance structures, and the experimental annotations from the DisProt database (19). Thus, these two predictors cover the main characteristics of the intrinsic disorder including the two types of disordered segments (short and long) and the three sources of the disorder annotations. The resulting five predictions were combined together using the majority vote-based consensus, motivated by the observation that consensus-based approaches provide improved predictive quality (18,20,21). Our use of the consensus-based approach is a marked improvement over the previous studies that utilized only one (22,23) or two (24,25) predictors to characterize disorder. This consensus-based approach was used to perform disorder prediction on the four considered complete proteomes: *H. sapiens*, *M. musculus*, *C. elegans* and *D. melanogaster*. The putative disorder was used to calculate the disorder content (i.e., fraction of disordered residues in a given chain), see Supporting Table S2.

Selection of Methods to Include in the Comparative Evaluation of DisoRDPbind

The prediction of RNA-binding (26,27) and DNA-binding (28) residues was pursued extensively over the last few years. However, these predictors focused on annotations of these binding events in ordered regions, i.e., that were annotated from crystal structures. These methods can be either structure-based (predictors that use protein structure as the input) or sequence-based. Table S4 lists recent (i.e., published after 2006) methods for the prediction of the ordered RNA- and DNA-binding residues. We note that DisoRDPbind focuses on different RNA- and DNA-binding regions that are located in IDRs. Since DisoRDPbind is the first method that predicts such binding events from the protein sequence, we compare it against representative sequence-based methods that predict ordered DNA- and RNA-binding residues, as the closest alternatives. We selected BindN+ (29), RNABindR v2.0 (30) and DNABR (31) (shown in bold in Table S4) to represent the predictors of ordered binding. BindN+ is a popular method that predicts both of RNA- and DNA-binging and was recently evaluated to provide accurate prediction (32). RNABindR v2.0 and DNABR are the latest sequence-based methods for the prediction of ordered RNA- and DNA-binding residues, respectively. The methods shown in italics in Table S4 are structure-based and thus could not be selected for the comparative analysis. Moreover, methods that do not provide real-valued probability outputs (propensity values) but only binary predictions (column “Prob. (Y/N)” set to “N” in Table S3), also could not be used.

We include the MoRFpred (15), DISOPRED3 (33) and ANCHOR (34,35) methods to compare predictions of the disordered protein-protein binding. We did not include PepBindPred (36) due to the relatively long runtime required for the molecular dynamics simulations used by this method.

Supporting Table S1. Definition of the RNA-, DNA-, and protein-binding and summary of the TRAINING, TEST115 and TEST36 datasets. The RNA-, DNA-, and protein-binding (2nd column) are defined by combining several functional subclasses listed in the 3rd column. The "Others" row (given in *italic*) includes all other functional subclasses which were not included in these datasets. The data were taken from release 6.01 of DisProt.

Dataset	Function	Functional subclass	# disordered residues
TRAINING	Protein-RNA binding	Protein-tRNA binding	308
		Protein-genomic RNA binding	435
		Protein-rRNA binding	971
		Protein-mRNA binding	319
		Protein-RNA binding	0
		**Total number**	**2033**
	Protein-DNA binding	Protein-DNA binding	5091
		DNA unwinding	90
		DNA bending	0
		**Total number**	**5146**
	Protein-protein binding	Protein-protein binding	22535
		Autoregulatory	1670
		Intraprotein interaction	1292
		Protein inhibitor	679
		Regulation of proteolysis in vivo	237
		**Total number**	**24290**
TEST114	Protein-RNA binding	Protein-tRNA binding	761
		Protein-genomic RNA binding	123
		Protein-rRNA binding	600
		Protein-mRNA binding	0
		Protein-RNA binding	387
		**Total number**	**1271**
	Protein-DNA binding	Protein-DNA binding	1420
		DNA unwinding	0
		DNA bending	102
		**Total number**	**1420**
	Protein-protein binding	Protein-protein binding	6689
		Autoregulatory	197
		Intraprotein interaction	208
		Protein inhibitor	48
		Regulation of proteolysis in vivo	0
		**Total number**	**6940**
TEST36	Protein-RNA binding	Protein-tRNA binding	42
		Protein-genomic RNA binding	0
		Protein-rRNA binding	0
		Protein-mRNA binding	0
		Protein-RNA binding	280
		**Total number**	**322**
	Protein-DNA binding	Protein-DNA binding	948
		DNA unwinding	0
		DNA bending	5
		**Total number**	**948**
	Protein-protein binding	Protein-protein binding	2634
		Autoregulatory	61
		Intraprotein interaction	1217
		Protein inhibitor	65
		Regulation of proteolysis in vivo	0
		**Total number**	**2752**
Other			*26501*

Supporting Table S2. Summary of the datasets extracted from the four considered complete genomes/proteomes: *H. sapiens*, *M. musculus*, *C. elegans* and *D. melanogaster*. The table shows the number of proteins and the average disorder content (fraction of disordered residues) for the proteins sets in the GO_RNA, GO_DNA, RBPDB, animalTFDB, DB_RNA, DB_DNA, ELM, and mentha datasets, where GO_RNA, RNPDB and DB_RNA include RNA-binding proteins; GO_DNA, animalTFDB and DB_DNA include DNA-binding proteins; mentha is the integrated source of protein-protein interaction (PPI) networks; and ELM includes motifs involved in the protein-protein interactions. The predRNA_UniProt, predDNA_UniProt, and predProtein_UniProt denotes the set of disordered DNA-, RNA-, and protein-binding proteins from UniProt, respectively, defined as those that have at least one disordered DNA-, RNA-, and protein-binding region (≥4 consecutive AAs) predicted by DisoRDPbind. For convenience, the ELMs located in the disordered regions are named disordered ELMs. A given ELM is defined as overlapping with the disordered protein-binding region (≥4 consecutive AAs) predicted by DisoRDPbind if there is at least one residue located in both of these two regions. PredProtein_mentha represents the predicted disordered protein-binding proteins from the mentha database. N/A indicates that a given species was not included in the corresponding dataset.

Species (taxID)	*H. sapiens* (9606)	*M. musculus* (10090)	*C. elegans* (6239)	*D. melanogaster* (7227)
# proteins collected from UniProt	42426	33181	25159	19656
% proteins with localization annotations	57%	72%	32%	44%
Average disorder content in UniProt	0.24	0.21	0.17	0.23
# proteins in GO_RNA	1209	1101	420	568
Average disorder content in GO_RNA	0.28	0.28	0.23	0.29
# proteins in RBPDB	398	339	204	73
Average disorder content in RBPDB	0.37	0.37	0.32	0.39
# proteins in DB_RNA	1068	802	N/A	N/A
Average disorder content in DB_RNA	0.33	0.32	N/A	N/A
# proteins in predRNA	2769	1401	722	792
Average disorder content in predRNA	0.37	0.34	0.32	0.35
# proteins in GO_DNA	3153	2686	1074	967
Average disorder content in GO_DNA	0.32	0.37	0.23	0.41
# proteins in animalTFDB	1464	1375	654	596
Average disorder in animalTFDB	0.32	0.34	0.22	0.41
# proteins in DB_DNA	677	126	N/A	N/A
Average disorder in DB_DNA	0.43	0.44	N/A	N/A
# proteins in predDNA	2475	2231	1241	1140
Average disorder content in predDNA	0.31	0.31	0.30	0.40
# proteins in mentha	14547	8006	5005	8096
Average number of interactors in mentha	21.4	6.7	5.2	7.3
# proteins in ELM	791	161	N/A	N/A
# ELMs in ELM	1242	206	N/A	N/A
# disordered ELMs in ELM	568	118	N/A	N/A
# disordered ELMs that overlap with the disordered protein-binding regions predicted by DisoRDPbind	539	115	N/A	N/A
# proteins in predProtein_UniProt	36150	28243	19683	17439
# proteins in predProtein_mentha	13525	7559	4553	7431

Supporting Table S3. Summary of the recent (developed after 2006) methods that predict ordered (annotated based on crystal structures) RNA- and DNA-binding residues. The methods for the prediction of the RNA- and DNA-binding are listed above and below the dash line, respectively. Each corresponding set of predictors is sorted by the year of publication. The "3-D" column indicates whether a given method performs predictions from 3-D structure or from sequence. The methods based on 3-D structure cannot be used to predict disordered RNA- and DNA-binding residues since structure cannot be provided for the intrinsically disordered regions. The "prob." and “binary” columns indicate whether it outputs probability (i.e., propensity score) and binary prediction, respectively. The “inactive" keyword in the “webserver URL” column indicates that we could not access a given webserver. Methods selected for comparative analysis are shown in bold. Methods shown in italics use structure for the prediction and thus could not be used in the comparative analysis.

Method (reference)	Year	3-D (Y/N)	Profile (Y/N)	Binary (Y/N)	Prob. (Y/N)	Webserver URL
**RNABindR v2.0 (30)**	**2012**	**N**	**Y**	**Y**	**Y**	**http://einstein.cs.iastate.edu/RNABindR/**
SPOT-Seq (37)	2011	N	Y	Y	N	http://sparks.informatics.iupui.edu/
SPOT-Stru (37)	*2011*	*Y*	*N*	*Y*	*N*	*http://sparks.informatics.iupui.edu/*
PRBR (38)	2011	N	Y	Y	Y	http://www.cbi.seu.edu.cn/PRBR/
**BindN+ (29)**	**2010**	**N**	**Y**	**Y**	**Y**	**http://bioinfo.ggc.org/bindn+/**
NAPS (39)	2010	N	Y	Y	Y	http://proteomics.bioengr.uic.edu/NAPS (inactive)
PRIP (40)	*2009*	*Y*	*Y*	*Y*	*Y*	*http://www.qfab.org/PRIP (inactive)*
PiRaNhA (41)	2009	N	Y	Y	Y	http://www.bioinformatics.sussex.ac.uk/PIRANHA (inactive)
Struct-NB (42)	*2008*	*Y*	*N*	*Y*	*Y*	*http://www.public.iastate.edu/~ftowfic (inactive)*
PPRINT (43)	2008	N	Y	Y	Y	http://www.imtech.res.in/raghava/pprint/
PRINTR (44)	2008	N	Y	Y	Y	http://210.42.106.80/printr/ (inactive)
BindN (45)	2006	N	N	Y	Y	http://bioinfo.ggc.org/bindn/
preDNA (46)	*2013*	*Y*	*Y*	*Y*	*Y*	*http://202.207.14.178/predna/index.aspx*
DR_bind (32)	*2012*	*Y*	*Y*	*Y*	*N*	*http://dnasite.limlab.ibms.sinica.edu.tw*
**DNABR (31)**	**2012**	**N**	**Y**	**Y**	**Y**	**http://www.cbi.seu.edu.cn/DNABR/**
metaDBsite (47)	2011	N	Y	Y	N	http://projects.biotec.tu-dresden.de/metadbsite/
DNABINDPROT (48)	*2010*	*Y*	*Y*	*Y*	*N*	*http://www.prc.boun.edu.tr/appserv/prc/dnabindprot/*
**BindN+ (29)**	**2010**	**N**	**Y**	**Y**	**Y**	**http://bioinfo.ggc.org/bindn+/**
NAPS (39)	2010	N	Y	Y	Y	http://proteomics.bioengr.uic.edu/NAPS
BindN-RF (49)	2009	N	Y	Y	Y	http://bioinfo.ggc.org/bindn-rf/
DISPLAR (50)	*2007*	*Y*	*Y*	*Y*	*N*	*http://pipe.scs.fsu.edu/displar.html*
Dp-bind (51)	2007	N	Y	Y	Y	http://lcg.rit.albany.edu/dp-bind/
DISIS (52)	2007	N	Y	Y	Y	http://cubic.bioc.columbia.edu/services/disis (inactive)
BindN (45)	2006	N	N	Y	Y	http://bioinfo.ggc.org/bindn/

Supporting Table S4. Fraction of known and putative binders with GO annotations of cellular component (cellular localization). The known DNA-binding (RNA-binding) proteins were collected from GO_DNA and animalTFDB (GO_RNA and RBPDB). The putative binders were predicted by DisoRDPbind and exclude the known binding proteins.

Protein type	Species	% of known binders	% of putative binders
RNA-binding	*H. sapiens*	80.6%	32.7%
	M. musculus	80.7%	61.9%
	C. elegans	58.3%	26.5%
	D. melanogaster	76.0%	37.4%
DNA-binding	*H. sapiens*	91.4%	56.6%
	M. musculus	93.8%	64.8%
	C. elegans	77.8%	30.2%
	D. melanogaster	77.1%	37.3%

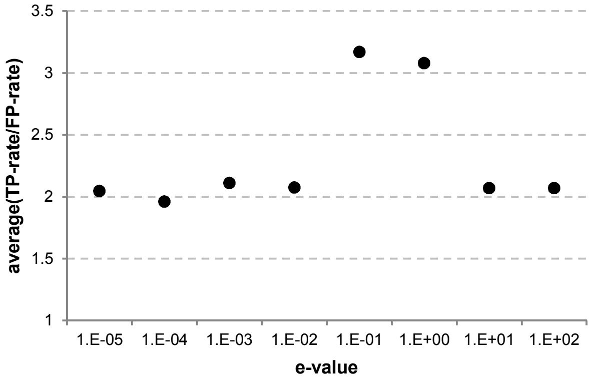

Supporting Figure S1. The predictive performance of BLAST in the function of the *e*-value thresholds based on the 4-fold cross-validation on the TRAINING dataset. The *y*-axis shows average (over the predictions of the disordered RNA-, DNA-, and protein-binding residues) value of the ratio of TP-rate and FP-rate; ratio>1 indicates good predictive performance, i.e., fraction of predicted true positives is higher compared to fraction of false positives.

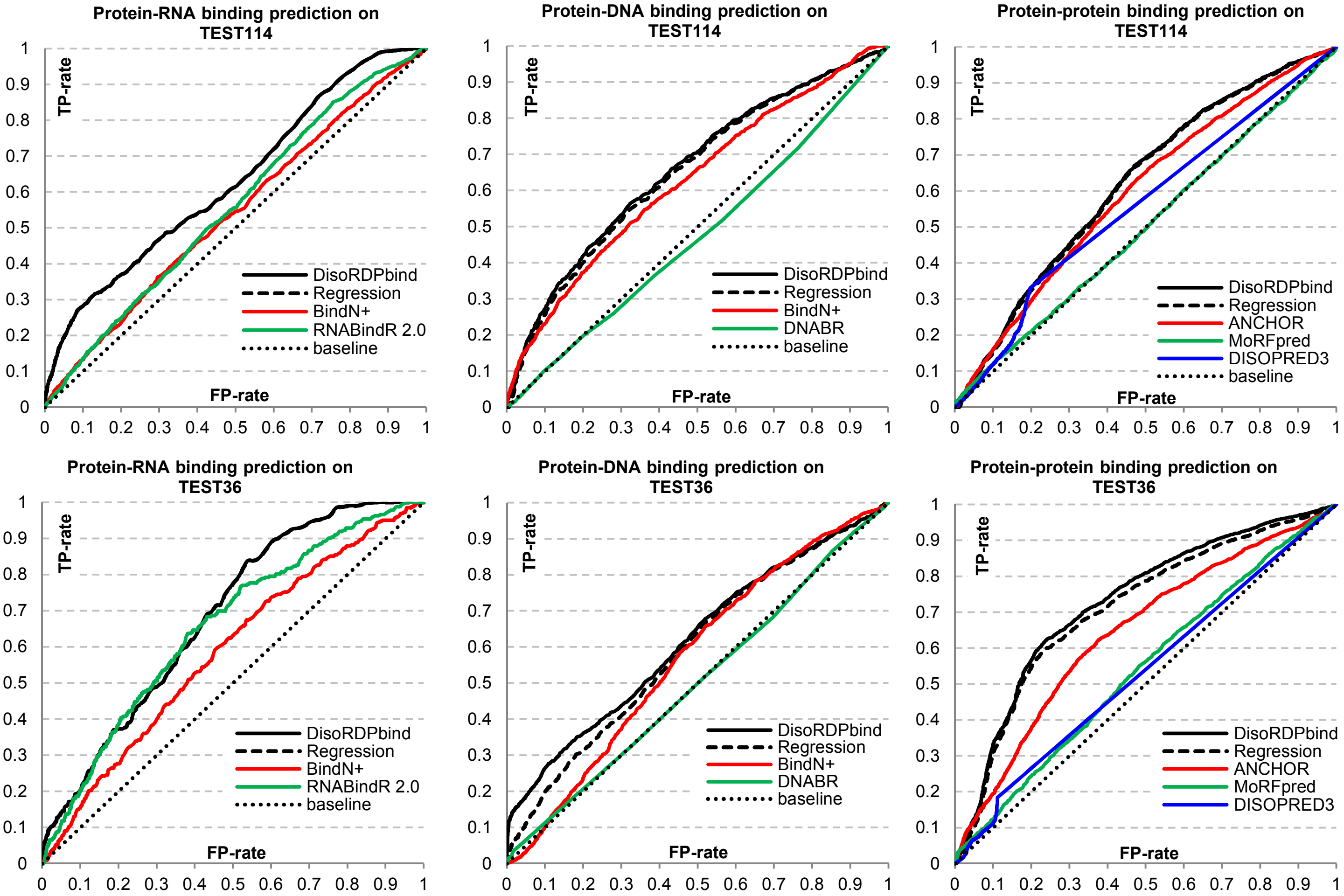

Supporting Figure S2. ROC curves for the prediction of the disordered RNA- (left most panels), DNA- (panels in the middle column), and protein-binding (right most panels) residues on the TEST114 datasets (top three panels) and the TEST36 dataset (lower three panels), respectively. Dotted black line denotes baseline, which corresponds to the results obtained with a random predictor. The “DisoRDP w/o BLAST” denotes DisoRDPbind without

the use of the BLAST-based alignment; we note that the ROC curves for the "DisoRDP w/o BLAST" in the left most panels overlap with the curves for DisoRDPbind.

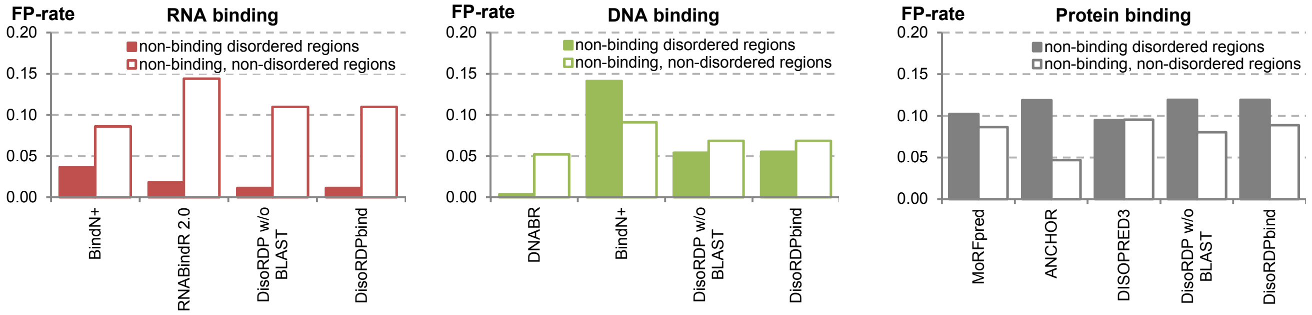

Supporting Figure S3. FP-rate for the prediction of the disordered RNA- (left panel), DNA- (middle panel), and protein-binding (right panel) residues on the two datasets of non-binding regions collected from the TEST114 dataset: disordered regions annotated with functions excluding a given binding type (non-binding disordered); and regions not located in disordered regions (non-binding, non-disordered). The "DisoRDP w/o BLAST" denotes DisoRDPbind without the use of the BLAST-based alignment.

REFERENCES

1. Kawashima, S., Ogata, H. and Kanehisa, M. (1999) AAindex: Amino Acid Index Database. *Nucleic Acids Research*, **27**, 368-369.
2. Kawashima, S. and Kanehisa, M. (2000) AAindex: amino acid index database. *Nucleic Acids Research*, **28**, 374.
3. Nakai, K., Kidera, A. and Kanehisa, M. (1988) Cluster analysis of amino acid indices for prediction of protein structure and function. *Protein Eng*, **2**, 93-100.
4. Tomii, K. and Kanehisa, M. (1996) Analysis of amino acid indices and mutation matrices for sequence comparison and structure prediction of proteins. *Protein Eng*, **9**, 27-36.
5. Anderson, T.W. and Darling, D.A. (1952) Asymptotic Theory of Certain Goodness of Fit Criteria Based on Stochastic Processes. *Ann Math Stat*, **23**, 193-212.
6. Wilcoxon, F. (1945) Individual Comparisons by Ranking Methods. *Biometrics Bulletin*, **1**, 80-83.
7. Rodgers, J.L. and Nicewander, W.A. (1988) 13 Ways to Look at the Correlation-Coefficient. *American Statistician*, **42**, 59-66.
8. Stigler, S.M. (1989) Francis Galton's Account of the Invention of Correlation. *Statistical Science*, **4**, 73-79.
9. Wootton, J.C. and Federhen, S. (1993) Statistics of Local Complexity in Amino-Acid-Sequences and Sequence Databases. *Comput Chem*, **17**, 149-163.
10. McGuffin, L.J., Bryson, K. and Jones, D.T. (2000) The PSIPRED protein structure prediction server. *Bioinformatics*, **16**, 404-405.
11. Dosztanyi, Z., Csizmok, V., Tompa, P. and Simon, I. (2005) IUPred: web server for the prediction of intrinsically unstructured regions of proteins based on estimated energy content. *Bioinformatics*, **21**, 3433-3434.

12. Dosztanyi, Z., Csizmok, V., Tompa, P. and Simon, I. (2005) The pairwise energy content estimated from amino acid composition discriminates between folded and intrinsically unstructured proteins. *J Mol Biol*, **347**, 827-839.
13. Tate, R.F. (1954) Correlation between a Discrete and a Continuous Variable - Point-Biserial Correlation. *Annals of Mathematical Statistics*, **25**, 603-607.
14. Kohavi, R. and John, G.H. (1997) Wrappers for feature subset selection. *Artificial Intelligence*, **97**, 273-324.
15. Disfani, F.M., Hsu, W.L., Mizianty, M.J., Oldfield, C.J., Xue, B., Dunker, A.K., Uversky, V.N. and Kurgan, L. (2012) MoRFpred, a computational tool for sequence-based prediction and characterization of short disorder-to-order transitioning binding regions in proteins. *Bioinformatics*, **28**, I75-I83.
16. Walsh, I., Martin, A.J., Di Domenico, T. and Tosatto, S.C. (2012) ESpritz: accurate and fast prediction of protein disorder. *Bioinformatics*, **28**, 503-509.
17. Peng, Z.L. and Kurgan, L. (2012) Comprehensive comparative assessment of in-silico predictors of disordered regions. *Curr Protein Pept Sci*, **13**, 6-18.
18. Walsh, I., Giollo, M., Di Domenico, T., Ferrari, C., Zimmermann, O. and Tosatto, S.C. (2015) Comprehensive large-scale assessment of intrinsic protein disorder. *Bioinformatics*, **31**, 201-208.
19. Sickmeier, M., Hamilton, J.A., LeGall, T., Vacic, V., Cortese, M.S., Tantos, A., Szabo, B., Tompa, P., Chen, J., Uversky, V.N. *et al.* (2007) DisProt: the Database of Disordered Proteins. *Nucleic Acids Res*, **35**, D786-793.
20. Peng, Z. and Kurgan, L. (2012) On the complementarity of the consensus-based disorder prediction. *Pac Symp Biocomput*, 176-187.
21. Fan, X. and Kurgan, L. (2014) Accurate prediction of disorder in protein chains with a comprehensive and empirically designed consensus. *Journal of biomolecular structure & dynamics*, **32**, 448-464.
22. Ward, J.J., Sodhi, J.S., McGuffin, L.J., Buxton, B.F. and Jones, D.T. (2004) Prediction and functional analysis of native disorder in proteins from the three kingdoms of life. *Journal of Molecular Biology*, **337**, 635-645.
23. Xue, B., Dunker, A.K. and Uversky, V.N. (2012) Orderly order in protein intrinsic disorder distribution: disorder in 3500 proteomes from viruses and the three domains of life. *J Biomol Struct Dyn*, **30**, 137-149.
24. Burra, P.V., Kalmar, L. and Tompa, P. (2010) Reduction in structural disorder and functional complexity in the thermal adaptation of prokaryotes. *PloS one*, **5**, e12069.
25. Xue, B., Williams, R.W., Oldfield, C.J., Dunker, A.K. and Uversky, V.N. (2010) Archaic chaos: intrinsically disordered proteins in Archaea. *BMC Syst Biol*, **4 Suppl 1**, S1.
26. Cirillo, D., Agostini, F. and Tartaglia, G.G. (2013) Predictions of protein–RNA interactions. *Wiley Interdisciplinary Reviews: Computational Molecular Science*, **3**, 161-175.
27. Puton, T., Kozlowski, L., Tuszynska, I., Rother, K. and Bujnicki, J.M. (2012) Computational methods for prediction of protein-RNA interactions. *J Struct Biol*, **179**, 261-268.
28. Kauffman, C. and Karypis, G. (2012) Computational tools for protein-DNA interactions. *Wires Data Min Knowl*, **2**, 14-28.
29. Wang, L., Huang, C., Yang, M. and Yang, J. (2010) BindN+ for accurate prediction of DNA and RNA-binding residues from protein sequence features. *BMC Syst Biol*, **4**, S3.
30. Walia, R.R., Caragea, C., Lewis, B.A., Towfic, F., Terribilini, M., El-Manzalawy, Y., Dobbs, D. and Honavar, V. (2012) Protein-RNA interface residue prediction using machine learning: an assessment of the state of the art. *BMC Bioinformatics*, **13**.
31. Ma, X., Guo, J., Liu, H.D., Xie, J.M. and Sun, X. (2012) Sequence-based prediction of DNA-binding residues in proteins with conservation and correlation information. *IEEE/ACM Trans Comput Biol Bioinform*, **9**, 1766-1775.

32. Chen, Y.C., Wright, J.D. and Lim, C. (2012) DR_bind: a web server for predicting DNA-binding residues from the protein structure based on electrostatics, evolution and geometry. *Nucleic Acids Res*, **40**, W249-256.
33. Jones, D.T. and Cozzetto, D. (2014) DISOPRED3: precise disordered region predictions with annotated protein-binding activity. *Bioinformatics*.
34. Dosztanyi, Z., Meszaros, B. and Simon, I. (2009) ANCHOR: web server for predicting protein binding regions in disordered proteins. *Bioinformatics*, **25**, 2745-2746.
35. Meszaros, B., Simon, I. and Dosztanyi, Z. (2009) Prediction of protein binding regions in disordered proteins. *PLoS computational biology*, **5**, e1000376.
36. Khan, W., Duffy, F., Pollastri, G., Shields, D.C. and Mooney, C. (2013) Predicting Binding within Disordered Protein Regions to Structurally Characterised Peptide-Binding Domains. *PloS one*, **8**.
37. Zhao, H., Yang, Y. and Zhou, Y. (2011) Highly accurate and high-resolution function prediction of RNA binding proteins by fold recognition and binding affinity prediction. *RNA Biol*, **8**, 988-996.
38. Ma, X., Guo, J., Wu, J., Liu, H., Yu, J., Xie, J. and Sun, X. (2011) Prediction of RNA-binding residues in proteins from primary sequence using an enriched random forest model with a novel hybrid feature. *Proteins*, **79**, 1230 - 1239.
39. Carson, M., Langlois, R. and Lu, H. (2010) NAPS: a residue-level nucleic acid-binding prediction server. *Nucleic Acids Res*, **38**, W431 - W435.
40. Maetschke, S. and Yuan, Z. (2009) Exploiting structural and topological information to improve prediction of RNA-protein binding sites. *BMC Bioinf*, **10**, 341.
41. Spriggs, R., Murakami, Y., Nakamura, H. and Jones, S. (2009) Protein function annotation from sequence: prediction of residues interacting with RNA. *Bioinformatics*, **25**, 1492 - 1497.
42. Towfic, F., Caragea, C., Gemperline, D., Dobbs, D. and Honavar, V. (2008) Struct-NB: predicting protein-RNA binding sites using structural features. *Int J Data Min Bioin*, **4**, 21 - 43.
43. Kumar, M., Gromiha, M. and Raghava, G. (2008) Prediction of RNA binding sites in a protein using SVM and PSSM profile. *Proteins*, **71**, 189 - 194.
44. Wang, Y., Xue, Z., Shen, G. and Xu, J. (2008) PRINTR: prediction of RNA binding sites in proteins using SVM and profiles. *Amino Acids*, **35**, 295 - 302.
45. Wang, L. and Brown, S. (2006) BindN: a web-based tool for efficient prediction of DNA and RNA binding sites in amino acid sequences. *Nucleic Acids Res*, **34 Web Server**, W243 - W248.
46. Li, T., Li, Q.Z., Liu, S., Fan, G.L., Zuo, Y.C. and Peng, Y. (2013) PreDNA: accurate prediction of DNA-binding sites in proteins by integrating sequence and geometric structure information. *Bioinformatics*, **29**, 678-685.
47. Si, J., Zhang, Z., Lin, B., Schroeder, M. and Huang, B. (2011) MetaDBSite: a meta approach to improve protein DNA-binding sites prediction. *BMC Syst Biol*, **5 Suppl 1**, S7.
48. Ozbek, P., Soner, S., Erman, B. and Haliloglu, T. (2010) DNABINDPROT: fluctuation-based predictor of DNA-binding residues within a network of interacting residues. *Nucleic Acids Res*, **38**, W417-423.
49. Wang, L., Yang, M.Q. and Yang, J.Y. (2009) Prediction of DNA-binding residues from protein sequence information using random forests. *BMC Genomics*, **10 Suppl 1**, S1.
50. Tjong, H. and Zhou, H.X. (2007) DISPLAR: an accurate method for predicting DNA-binding sites on protein surfaces. *Nucleic Acids Res*, **35**, 1465-1477.
51. Hwang, S., Gou, Z. and Kuznetsov, I.B. (2007) DP-Bind: a web server for sequence-based prediction of DNA-binding residues in DNA-binding proteins. *Bioinformatics*, **23**, 634-636.
52. Ofran, Y., Mysore, V. and Rost, B. (2007) Prediction of DNA-binding residues from sequence. *Bioinformatics*, **23**, i347 - i353.
